# Quality of maternal and newborn care in Switzerland during the COVID‐19 pandemic: A cross‐sectional study based on WHO quality standards

**DOI:** 10.1002/ijgo.14456

**Published:** 2022-12-18

**Authors:** Claire de Labrusse, Alessia Abderhalden‐Zellweger, Ilaria Mariani, Anouck Pfund, Michael Gemperle, Susanne Grylka‐Baeschlin, Antonia N. Mueller, Emanuelle Pessa Valente, Benedetta Covi, Marzia Lazzerini, the IMAgiNE EURO study group, Amira Ćerimagić, Daniela Drandić, Magdalena Kurbanović, Rozée Virginie, Elise de La Rochebrochard, Kristina Löfgren, Céline Miani, Stephanie Batram‐Zantvoort, Lisa Wandschneider, Marzia Lazzerini, Emanuelle Pessa Valente, Benedetta Covi, Ilaria Mariani, Sandra Morano, Ilana Chertok, Emek Hefer, Rada Artzi‐Medvedik, Elizabete Pumpure, Dace Rezeberga, Gita Jansone‐Šantare, Dārta Jakovicka, Anna Regīna Knoka, Katrīna Paula Vilcāne, Alina Liepinaitienė, Andželika Kondrakova, Marija Mizgaitienė, Simona Juciūtė, Maryse Arendt, Barbara Tasch, Ingvild Hersoug Nedberg, Sigrun Kongslien, Eline Skirnisdottir Vik, Barbara Baranowska, Urszula Tataj‐Puzyna, Maria Węgrzynowska, Raquel Costa, Catarina Barata, Teresa Santos, Carina Rodrigues, Heloísa Dias, Marina Ruxandra Otelea, Jelena Radetić, Jovana Ružičić, Zalka Drglin, Barbara Mihevc Ponikvar, Anja Bohinec, Serena Brigidi, Lara Martín Castañeda, Helen Elden, Verena Sengpiel, Karolina Linden, Mehreen Zaigham, Claire De Labrusse, Alessia Abderhalden, Anouck Pfund, Harriet Thorn, Susanne Grylka, Michael Gemperle, Antonia Mueller

**Affiliations:** ^1^ School of Health Sciences (HESAV) HES‐SO University of Applied Sciences and Arts Western Switzerland Lausanne Switzerland; ^2^ WHO Collaborating Center for Maternal and Child Health Institute for Maternal and Child Health IRCCS “Burlo Garofolo” Trieste Italy; ^3^ Research Institute for Midwifery ZHAW Zurich University of Applied Sciences Winterthur Switzerland

**Keywords:** COVID‐19, IMAgiNE EURO, maternal health, maternity services, mode of birth, quality of care, Switzerland, WHO standards

## Abstract

**Objective:**

To explore quality of maternal and newborn care (QMNC) in healthcare facilities during the COVID‐19 pandemic in Switzerland.

**Methods:**

Women giving birth in Switzerland answered a validated online questionnaire including 40 WHO standards‐based quality measures. QMNC score was calculated according to linguistic region and mode of birth. Differences were assessed using logistic regression analysis adjusting for relevant variables.

**Results:**

A total of 1175 women were included in the analysis. Limitations in QMNC during the pandemic were reported by 328 (27.9%) women. Several quality measures, such as deficient communication (18.0%, *n* = 212), insufficient number of healthcare professionals (19.7%, *n* = 231), no information on the newborn after cesarean (26.5%, *n* = 91) or maternal and newborn danger signs (34.1%, *n* = 401 and 41.4% *n* = 487, respectively) suggested preventable gaps in QMNC. Quality measures significantly differed by linguistic region and mode of birth. Multivariate analysis established a significantly lower QMNC for women in French‐ and Italian‐speaking regions compared with the German‐speaking region. Moreover, in several quality indicators reflecting communication with healthcare providers, women who did not answer the questionnaire in one of the Swiss national languages had significantly worse scores than others. A significant lower QMNC was also found for young and primiparous women and for those who experienced cesarean or instrumental vaginal birth.

**Conclusion:**

Women giving birth in Switzerland during the pandemic reported notable gaps in QMNC. Providers should be attuned to women who are younger, primiparous, and those who had an emergency cesarean or instrumental vaginal birth given the lower QMNC reported by these groups. Women who did not respond in a Swiss national language may need improved communication strategies.

## Synopsis

Women giving birth in Switzerland during the COVID‐19 pandemic reported several shortcomings in quality of maternal and newborn care. Substantial differences across linguistic regions and mode of birth were observed.

## 1 INTRODUCTION

The World Health Organization (WHO) recognizes the need for every woman to receive high‐quality maternal and newborn care throughout pregnancy, childbirth, and the postnatal period.^1^ Poor quality of maternal and newborn care (QMNC) can have an impact on preventable maternal, fetal, and newborn morbidity and mortality by increasing the risks of pregnancy‐ and childbirth‐related complications.^2^ The COVID‐19 pandemic shattered many aspects of daily life, as drastic measures such as quarantine, isolation, and other restrictions were implemented to slow down transmission of the virus. Several international organizations, such as the International Confederation of Midwives and the WHO, stressed the importance of providing pregnant women with high‐quality and respectful care even during unexpected circumstances,^3^, ^4^ in accordance with a woman‐centered care approach.^5^ However, international literature shows that the quality of maternal and newborn care provided has been greatly affected during the COVID‐19 pandemic.^6^ For example, studies reported a reduction of in‐person visits, fewer antenatal appointments or emergency care admissions for pregnant women, and an over‐medicalization of perinatal care (e.g. cesarean, induction of labor).^7^, ^8^, ^9^ Additionally, as the involvement of a woman's partner and family were reduced due to restrictions put in place by maternity services, issues related to scarce social support have been documented among women.^10^, ^11^, ^12^

On March 16, 2020, Switzerland declared a National State of Emergency, which meant that the usually highly independent cantons had to give up many of their decision‐making powers to the central government.^13^ Preventive measures such as border closures, event restrictions, and closure of schools, restaurants, bars, and shops were uniformly implemented to slow down and prevent the spread of COVID 19.^13^ In Switzerland, three main peaks are evident since March 2020: (1) from October 2020 to February 2021; (2) from August 2021 to October 2021; and (3) from November 2021 to March 2022.^14^

Specific measures and national recommendations within maternity services were issued by the Swiss Society of Gynecology and Obstetrics and the Swiss Federation of Midwives during the COVID 19 pandemic.^15^, ^16^ For example, unlike before, visits by family members were limited or even prohibited, the partner could accompany the woman in labor but could only remain for a short time after birth, and face‐to‐face care by healthcare professionals (HCPs) was reduced.

No known peer‐reviewed published information documenting the impact of the COVID‐19 pandemic on QMNC in Switzerland was evident from a literature search. The present study was conducted as a part of the IMAgiNE EURO study to describe the QMNC of women who gave birth in Switzerland during the COVID‐19 pandemic, as well as variations in the reported QMNC according to linguistic region and mode of birth. The aim of the IMAgiNE EURO study was to understand the views and experiences of women giving birth, as well as health workers involved in maternal and newborn care among 18 European countries.^17^

## 2 MATERIALS AND METHODS

### 2.1 Study design

IMAgiNE EURO is a cross‐sectional study conducted in several European countries based on a validated online questionnaire.^18^ The study was registered in ClinicalTrials.gov (NCT04847336). For the reporting of this study, the STROBE guidelines for observational studies were used.^19^

### 2.2 Study setting

Switzerland is divided into 26 cantons that can be grouped into three major linguistic regions (German‐speaking, French‐speaking, and Italian‐speaking): (1) Espace Mittelland, Eastern Switzerland, and Central Switzerland where 75% of the population speaks German; (2) Western Switzerland where 78% of the population speaks French; and (3) Ticino where 88% of the population speaks Italian.^20^ The Swiss health system is characterized by a decentralized mode of operation, where the 26 cantons are mainly responsible for all health‐related legislations and regulations, and the Federal Council essentially figures as mediator of cantonal health policies.^21^

### 2.3 Study population

The present study included women who gave birth from March 1, 2020, up to February 7, 2022, in hospitals and clinics established in the three main linguistic regions of Switzerland. Women aged 18 years and older who spoke one of the 24 languages available in the questionnaire were eligible to participate.

### 2.4 Sample size estimation

**FIGURE 1:**
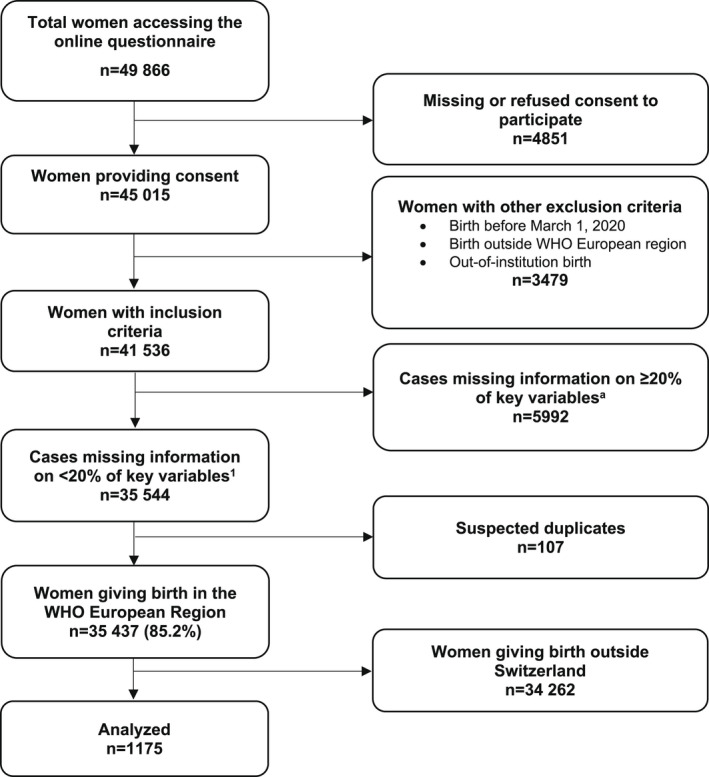
Study flow diagram. ^a^We used 45 key variables (40 key quality measures and five key sociodemographic questions).

A minimum sample size of 100 women per main national language (German, French, and Italian) was estimated to be adequate to detect a minimum frequency on each quality measure of 4% ± 4%, with a confidence level of 96%. We assumed that women answering in a national language lived in the corresponding linguistic region.^20^ The language in which women responded to the survey was therefore used as a proxy for linguistic region. Cases were excluded from the primary analysis if they were suspected duplicates (Figure 1) or had 20% or more missing values for the 45 key variables (40 quality measures and five key sociodemographic variables such as year of birth, age, education, parity, and whether or not the woman gave birth in the same country where she was born).

### 2.5 Study procedure

A predefined dissemination plan was used to promote the survey. Women were recruited using various approaches such as mother‐centered and parental groups on social media platforms or by distributing flyers to healthcare institutions and via local networks. A link and QR code were available within the message which redirected the participant to the online questionnaire.

### 2.6 Data collection tool

Data were collected using a validated questionnaire^18^ containing 40 items (one for each quality measure) based on the standards for improving QMNC in health facilities defined by the WHO.^1^ These items were evenly dispersed across four domains: (1) provision of care; (2) experience of care; (3) availability of human and physical resources; and (4) key organizational changes related to the COVID‐19 pandemic. The 40 quality measures are listed in supporting information Table 1 and were used to generate a QMNC index, with higher scores indicating greater adherence to WHO standards. Development, validation, and previous use of the questionnaire have been reported elsewhere.^18^

### 2.7 Data analysis

Descriptive analysis was conducted computing absolute and relative frequencies for each categorical variable. Two subgroup analyses were performed: by linguistic region (i.e. French‐, Italian‐, German‐speaking region, and women who answered the questionnaire in other languages) and by mode of birth (i.e. vaginal birth, instrumental vaginal birth [IVB], and emergency cesarean). Women who underwent emergency cesarean were also assigned to a group based on whether they did or did not experience labor, according to the NICE definition of labor,^22^ which was included in the questionnaire. Differences between linguistic regions and mode of birth were assessed through logistic regression analysis adjusting for relevant variables (i.e. maternal age, maternal education, year of birth, women born in Switzerland, type of facility, parity, presence of an obstetrics/gynecology doctor at birth, multiple birth, mother's admission to intensive care, and newborn admission to neonatal intensive care or a special care baby unit). Adjusted *P* values from the logistic regression model were reported for each quality measure.

To assess robustness of descriptive findings, two sensitivity analyses were conducted: (1) including only women who answered 100% of the 45 key variables; and (2) including women with up to 90% missing values for the 45 key variables, as previously done by other authors of surveys.^23^

A QMNC index was calculated based on predefined criteria for all cases with complete quality measures (supporting information Table 2). In each of the four domains, the QMNC index could range from 0 to 100. Thus, the total range was from 0 to 400. The QMNC indexes were not normally distributed and therefore are presented as median and interquartile ranges.

To study whether the QMNC index differed between linguistic regions or mode of birth, multivariate logistic regression analysis was used to identify any association between the variable of interest (i.e. linguistic region, mode of birth) with the dependent variable (i.e. QMNC index) by adjusting for the other relevant variables associated with the dependent variable (i.e. maternal age, maternal education, year of birth, woman born in Switzerland, type of facility, parity, presence of an obstetrics/gynecology doctor at birth). Multivariate quantile regression with robust standard errors was used, modeling the median and the 0.25th and 0.75th quantiles because of statistical evidence of heteroskedasticity (Breusch‐Pagan/Cook‐Weisberg test *P* < 0.001 H0: homoskedasticity).^24^ The reference categories were determined to be the ones with the highest frequencies.

Statistical significance was established at a two‐tailed *P* value of <0.05. Stata Version 14 (StataCorp LLC) and R (version 4.1.1)^25^ were used for all statistical analyses.

## 3 RESULTS

Of the 49 866 women who accessed the IMAgiNE EURO online questionnaire, 41 536 met the inclusion criteria. Among them, 1175 were included in the present analysis, after removing all women who had given birth outside of Switzerland, cases missing 20% or more of key variables, and suspected duplicates (Figure 1). Strong connections with health institutions and local network providers enabled us to reach the sample size estimation in every linguistic region.

### 3.1 Characteristics of the study population

Characteristics of the respondents are detailed in Table 1. Most women (90.1%, *n* = 1059) were aged 25–39 years, and 90.3% (*n* = 1061) had finished high school. Half of the participants (50.8%, *n* = 597) had had a previous birth, and about three‐quarters (75.3%, *n* = 885) gave birth in a public hospital. More than a quarter (27.8%, *n* = 327) were born outside of Switzerland, and 91.9% (*n* = 1080) answered the questionnaire in one of the national languages (47.0% [*n* = 552] in French, 33.1% [*n* = 389] in German, 11.8% [*n* = 139] in Italian, and 8.1% [*n* = 95] in another language).

**TABLE 1:** Characteristics of respondents.

Characteristics	No. (%) (*n* = 1175)
Year/date of birth	
2020	582 (49.5)
2021	558 (47.5)
2022	8 (0.7)
Missing	27 (2.3)
Mother born in Switzerland	
Yes	825 (70.2)
No	327 (27.8)
Missing	23 (2.0)
Age range, years	
18–24	16 (1.4)
25–30	257 (21.9)
31–35	555 (47.2)
36–39	247 (21.0)
≥40	77 (6.6)
Missing	23 (2.0)
Educational level^a^	
None	0 (0.0)
Elementary school	13 (1.1)
Junior High school	78 (6.6)
High School	252 (21.4)
University degree	394 (33.5)
Postgraduate degree/Masters/Doctorate or higher	415 (35.3)
Missing	23 (2.0)
Language of the questionnaire	
Albanian	1 (0.1)
Bosnian	2 (0.2)
Croatian	2 (0.2)
English	54 (4.6)
French	552 (47.0)
German	389 (33.1)
Italian	139 (11.8)
Latvian	4 (0.3)
Norwegian	1 (0.1)
Polish	5 (0.4)
Portuguese	13 (1.1)
Romanian	5 (0.4)
Spanish	8 (0.7)
Parity	
1	555 (47.2)
>1	597 (50.8)
Missing	23 (2.0)
Birth mode	
Spontaneous vaginal birth	698 (59.4)
Instrumental vaginal birth	134 (11.4)
Cesarean	343 (29.2)
Emergency cesarean during labor	111 (9.4)
Emergency cesarean before labor	67 (5.7)
Planned or elective cesarean before labor	165 (14.0)
Other clinical characteristics	
Baby admitted to neonatal intensive care unit or special care baby unit	92 (7.8)
Mother admitted to intensive care unit	10 (0.9)
Stillbirth	1 (0.1)
Multiple birth	34 (2.9)
Type of hospital	
Public	885 (75.3)
Private	267 (22.7)
Missing	23 (2.0)
Type of healthcare provider who directly assisted birth	
Midwife or nurse	1103 (93.9)
Student (i.e. before graduation)	204 (17.4)
Obstetrics registrar/medical resident (under postgraduate training)	250 (21.3)
Obstetrics/gynecology doctor	861 (73.3)
Unknown (healthcare providers did not introduce themselves)	51 (4.3)
Other	98 (8.3)

^a^ Wording on education levels agreed among partners during the Delphi. Questionnaire translated and back‐translated according to ISPOR Task Force for Translation and Cultural Adaptation Principles of Good Practice.

More than half of the women (59.4%, *n* = 698) had a spontaneous vaginal birth (SVB), 11.4% (*n* = 134) an IVB, and 29.2% (*n* = 343) a cesarean, half of which were elective. Most women were assisted during birth by a midwife or a nurse (93.9%, *n* = 1103), and almost three‐quarters (73.3%, *n* = 861) reported the presence of an obstetrics/gynecology doctor.

### 3.2 Key quality measures of QMNC by linguistic region

In the provision of care domain of the questionnaire, data showed significant differences for several items according to linguistic regions (Table 2). Regarding mode of birth, 9.4% (*n* = 111) of women had an emergency cesarean after labor started (6.5% [*n* = 36] in the French‐speaking region to 12.9% [*n* = 18] in the Italian‐speaking region, *P* = 0.001). In total, 19.3% (*n* = 227) of women did not receive immediate attention when needed (12.1% [*n* = 47] in the German‐speaking region to 25.2% [*n* = 139] in the French‐speaking region, *P* < 0.001).

**TABLE 2:** Key quality measures of QMNC according to linguistic region^a^ ^,^ ^b^ ^,^ ^c^

Key quality measures of QMNC (40 items)		Overall	Women in French‐speaking region	Women in German‐speaking region	Women in Italian‐speaking region	Women who answered the questionnaire in other languages	adj *P* value^d^
		No. (%)	No. (%)	No. (%)	No. (%)	No. (%)	
No.		1175	552	389	139	95	
Provision of care							
1	No pain relief during labor (for SVB, IVB, emergency cesarean during labor)	134/943 (14.2)	46/438 (10.5)	62/322 (19.3)	19/111 (17.1)	7/72 (9.7)	**0.001**
2a	Mode of birth: instrumental vaginal birth	134 (11.4)	61 (11.1)	42 (10.8)	18 (12.9)	13 (13.7)	>0.99
2b	Mode of birth: emergency cesarean during labor	111 (9.4)	36 (6.5)	45 (11.6)	18 (12.9)	12 (12.6)	**0.001**
2c	Mode of birth: emergency cesarean before labor	67 (5.7)	37 (6.7)	15 (3.9)	9 (6.5)	6 (6.3)	0.283
2d	Mode of birth: planned or elective cesarean	165 (14.0)	77 (13.9)	52 (13.4)	19 (13.7)	17 (17.9)	0.052
3a	Episiotomy (in SVB)	64/698 (9.2)	29/341 (8.5)	21/235 (8.9)	5/75 (6.7)	9/47 (19.1)	0.449
3b	Fundal pressure (in IVB)	22/134 (16.4)	9/61 (14.8)	6/42 (14.3)	5/18 (27.8)	2/13 (15.4)	0.645
3c	No pain relief after cesarean	31/343 (9.0)	19/150 (12.7)	9/112 (8.0)	1/46 (2.2)	2/35 (5.7)	0.163
4	No skin to skin	60 (5.1)	40 (7.2)	8 (2.1)	10 (7.2)	2 (2.1)	**0.002**
5	No early breastfeeding	119 (10.1)	68 (12.3)	26 (6.7)	18 (12.9)	7 (7.4)	**0.013**
6	Inadequate breastfeeding support	197 (16.8)	104 (18.8)	42 (10.8)	33 (23.7)	18 (18.9)	**0.001**
7	No rooming‐in	200 (17.0)	95 (17.2)	56 (14.4)	33 (23.7)	16 (16.8)	**0.029**
8	Not allowed to stay with the baby as wished	37 (3.1)	23 (4.2)	6 (1.5)	2 (1.4)	6 (6.3)	**0.014**
9	No exclusive breastfeeding at discharge	278 (23.7)	127 (23.0)	83 (21.3)	41 (29.5)	27 (28.4)	0.282
10	No immediate attention when needed	227 (19.3)	139 (25.2)	47 (12.1)	23 (16.5)	18 (18.9)	**<0.001**
Experience of care							
1a	No freedom of movements during labor	90/943 (9.5)	42/438 (9.6)	20/322 (6.2)	18/111 (16.2)	10/72 (13.9)	0.053
1b	No consent requested for vaginal examination in prelabor cesarean	20/232 (8.6)	9/114 (7.9)	8/67 (11.9)	3/28 (10.7)	0/23 (0.0)	0.589
2a	No choice of birth position (in SVB)	238/698 (34.1)	129/341 (37.8)	68/235 (28.9)	21/75 (28.0)	20/47 (42.6)	0.078
2b	No consent requested (for IVB)	54/134 (40.3)	27/61 (44.3)	16/42 (38.1)	7/18 (38.9)	4/13 (30.8)	0.723
2c	No information on newborn after cesarean	91/343 (26.5)	59/150 (39.3)	18/112 (16.1)	10/46 (21.7)	4/35 (11.4)	**<0.001**
3	No clear/effective communication from HCP	212 (18.0)	132 (23.9)	34 (8.7)	24 (17.3)	22 (23.2)	**<0.001**
4	No involvement in choices	245 (20.9)	147 (26.6)	51 (13.1)	28 (20.1)	19 (20.0)	**<0.001**
5	Companionship not allowed	398 (33.9)	179 (32.4)	137 (35.2)	46 (33.1)	36 (37.9)	0.574
6	Not treated with dignity	145 (12.3)	77 (13.9)	41 (10.5)	14 (10.1)	13 (13.7)	0.279
7	No emotional support	229 (19.5)	117 (21.2)	68 (17.5)	24 (17.3)	20 (21.1)	0.653
8	No privacy	116 (9.9)	64 (11.6)	32 (8.2)	11 (7.9)	9 (9.5)	0.346
9	Abuse (physical/verbal/emotional)	113 (9.6)	60 (10.9)	32 (8.2)	12 (8.6)	9 (9.5)	0.519
10	Informal payment	39 (3.3)	21 (3.8)	11 (2.8)	4 (2.9)	3 (3.2)	0.844
Availability of physical and human resources							
1	No timely care by HCP at facility arrival	92 (7.8)	47 (8.5)	28 (7.2)	11 (7.9)	6 (6.3)	0.669
2	No information on maternal danger signs	401 (34.1)	220 (39.9)	105 (27.0)	42 (30.2)	34 (35.8)	**0.001**
3	No information on newborn danger signs	487 (41.4)	250 (45.3)	133 (34.2)	59 (42.4)	45 (47.4)	**0.007**
4	Inadequate room comfort and equipment	11 (0.9)	8 (1.4)	3 (0.8)	0 (0.0)	0 (0.0)	0.738
5	Inadequate number of women per rooms	61 (5.2)	31 (5.6)	18 (4.6)	2 (1.4)	10 (10.5)	0.144
6	Inadequate room cleaning	10 (0.9)	5 (0.9)	3 (0.8)	1 (0.7)	1 (1.1)	0.942
7	Inadequate bathroom	24 (2.0)	17 (3.1)	4 (1.0)	2 (1.4)	1 (1.1)	0.320
8	Inadequate partner visiting hours	289 (24.6)	159 (28.8)	81 (20.8)	25 (18.0)	24 (25.3)	**0.035**
9	Inadequate HCP number	112 (9.5)	60 (10.9)	30 (7.7)	13 (9.4)	9 (9.5)	0.214
10	Inadequate HCP professionalism	18 (1.5)	11 (2.0)	4 (1.0)	2 (1.4)	1 (1.1)	0.507
Reorganizational changes due to COVID‐19							
1	Difficulties in attending routine antenatal visits	238 (20.3)	114 (20.7)	85 (21.9)	22 (15.8)	17 (17.9)	0.519
2	Any barriers in accessing the facility	183 (15.6)	80 (14.5)	73 (18.8)	21 (15.1)	9 (9.5)	0.456
3	Inadequate infographics	189 (16.1)	81 (14.7)	42 (10.8)	38 (27.3)	28 (29.5)	**<0.001**
4	Inadequate wards reorganization	250 (21.3)	119 (21.6)	79 (20.3)	27 (19.4)	25 (26.3)	0.878
5	Inadequate room reorganization	324 (27.6)	153 (27.7)	102 (26.2)	44 (31.7)	25 (26.3)	0.342
6	Lacking one functioning accessible hand‐washing station	42 (3.6)	18 (3.3)	12 (3.1)	12 (8.6)	0 (0.0)	0.148
7	HCP not always using PPE	100 (8.5)	49 (8.9)	34 (8.7)	9 (6.5)	8 (8.4)	0.832
8	Insufficient HCP number	231 (19.7)	135 (24.5)	52 (13.4)	31 (22.3)	13 (13.7)	**<0.001**
9	Communication inadequate to contain COVID‐19‐related stress	255 (21.7)	139 (25.2)	66 (17.0)	25 (18.0)	25 (26.3)	**0.004**
10	Reduction in QMNC due to COVID‐19	328 (27.9)	149 (27.0)	109 (28.0)	39 (28.1)	31 (32.6)	0.779

Abbreviations: HCP, healthcare professional; IVB, instrumental vaginal birth; PPE, personal protective equipment; QMNC, quality of maternal and newborn care; SVB, spontaneous vaginal birth.

^a^ All the indicators in the domains of provision of care, experience of care, and resources are directly based on WHO standards.

^b^ Indicators with a specified denominator (e.g. 3a, 3b) were tailored to take into account different modes of birth (i.e. spontaneous vaginal, instrumental vaginal, and cesarean). These were calculated on subsamples (e.g. 3a was calculated on spontaneous vaginal births; 3b was calculated on instrumental vaginal births).

^c^ Indicator 6 in the domains of reorganizational changes due to COVID‐19 was defined as: at least one functioning and accessible hand‐washing station (near or inside the room where the mother was hospitalized) supplied with water and soap or with disinfectant alcohol solution.

^d^ *P* values were obtained from the logistic regression model testing quality measures difference by linguistic region adjusted for sociodemographic and obstetric variables (i.e. maternal age, education, year of birth, mode of birth, parity, presence of an obstetrician/gynecologist at birth, multiple birth), type of hospital, newborn admission to neonatal intensive care or special care baby unit, and mother's admission to intensive care. Bold values are statistically significant.

For the domain of experience of care, significant differences across linguistic regions were observed. More than a quarter of women (26.5%, *n* = 91) reported receiving no information on their newborn after cesarean (from 16.1% [*n* = 18] in the German‐speaking region to 39.3% [*n* = 59] in the French‐speaking region, *P* < 0.001). About one in five women (18.0%, *n* = 212) complained about no clear/effective communication from HCPs (from 8.7% [*n* = 34] in the German‐speaking region to 23.9% [*n* = 132] in the French‐speaking region, *P* < 0.001). Furthermore, 20.9% (*n* = 245) did not feel involved in choices related to the medical interventions they received (13.1% [*n* = 51] in the German‐speaking region to 26.6% [*n* = 147] for women in the French‐speaking region, *P* < 0.001). Overall, 33.9% (*n* = 398) of women mentioned that their companion was not allowed to be present during birth and 19.5% (*n* = 229) reported an absence of emotional support around the time of childbirth.

In the domain of availability of physical and human resources, 34.1% (*n* = 401) of women mentioned that they received no information on maternal danger signs (from 27.0% [*n* = 105] in the German‐speaking region to 39.9% [*n* = 220] in the French‐speaking region, *P* = 0.001), and 41.4% (*n* = 487) received no information on newborn danger signs (from 34.2% [*n* = 133] in the German‐speaking region to 45.3% [*n* = 250] in the French‐speaking region, *P* = 0.007). Almost half (47.4%, *n* = 45) of women answering in other languages also perceived not receiving information on newborn danger signs. Finally, 24.6% (*n* = 289) of women felt that the visiting hours for partners/relatives were inadequate (from 18.0% [*n* = 25] in the Italian‐speaking region to 28.8% [*n* = 159] in the French‐speaking region, *P* = 0.035).

Key QMNC indicators in the domain of organizational changes due to the COVID‐19 pandemic showed that more than one in four women (27.9%, *n* = 328) reported a reduction in QMNC due to COVID‐19. Difficulties in attending routine antenatal visits during the pandemic were reported by 20.3% (*n* = 238) of women, 21.3% (*n* = 250) disclosed an inadequacy in ward reorganization, and 27.6% (*n* = 324) an inadequacy in rooms organization (to reduce the risk of infection). Some women (16.1%, *n* = 189) reported inadequate infographics, such as posters and images, to indicate the path or the rules to follow to reduce the risk of infection as much as possible (from 10.8% [*n* = 42] in the German‐speaking region to 27.3% [*n* = 38] in the Italian‐speaking region, *P* < 0.001). Women who answered in other languages were also particularly affected by this problem (29.5%, *n* = 28). An insufficient number of HCPs to guarantee adequate assistance despite the COVID‐19 pandemic was reported by 19.7% (*n* = 231) of women (from 13.4% [*n* = 52] in the German‐speaking region to 24.5% [*n* = 135] in the French‐speaking region, *P* < 0.001). Finally, 21.7% (*n* = 255) revealed that the communication received was inadequate to reduce COVID‐19‐related stress (from 17.0% [*n* = 66] in the German‐speaking region to 25.2% [*n* = 139] in the French‐speaking region). Lack of adequate communication was also reported by 26.3% (*n* = 25) of women who responded in other languages.

### 3.3 Key quality measures of QMNC by mode of birth

In the domain of provision of care (Table 3), lack of early breastfeeding was reported by 5.2% (*n* = 36) of women with SVB compared with 23.6% (*n* = 39) of women with elective cesarean. In addition, no exclusive breastfeeding at discharge was reported by 17.6% (*n* = 123) women with SVB compared with 37.3% (*n* = 25) of women with emergency cesarean before labor (*P* < 0.001).

**TABLE 3:** Key quality measures of QMNC by mode of birth^a^ ^,^ ^b^ ^,^ ^c^

Key quality measures of QMNC (40 items)		Spontaneous vaginal birth	Instrumental vaginal birth	Emergency cesarean during labor	Emergency cesarean before labor	Planned or elective cesarean	adj *P* value^d^
		No. (%) (*n* = 698)	No. (%) (*n* = 134)	No. (%) (*n* = 111)	No. (%) (*n* = 67)	No. (%) (*n* = 165)	
Provision of care							
1	No pain relief during labor (for SVB, IVB, emergency cesarean during labor)	122 (17.5)	5 (3.7)	7 (6.3)			**<0.001**
2a	Mode of birth: Instrumental vaginal birth	‐	134 (100.0)	‐	‐	‐	‐
2b	Mode of birth: emergency cesarean during labor	‐	‐	111 (100.0)	‐	‐	‐
2c	Mode of birth: Emergency cesarean before labor	‐	‐	‐	67 (100.0)	‐	‐
2d	Mode of birth: Planned or elective cesarean	‐	‐	‐	‐	165 (100.0)	‐
3a	Episiotomy (in SVB)	64 (9.2)	‐	‐	‐	‐	‐
3b	Fundal pressure (in IVB)	‐	22 (16.4)	‐	‐	‐	‐
3c	No pain relief after cesarean	‐	‐	9 (8.1)	10 (14.9)	12 (7.3)	0.598
4	No skin to skin	7 (1.0)	3 (2.2)	21 (18.9)	10 (14.9)	19 (11.5)	**<0.001**
5	No early breastfeeding	36 (5.2)	15 (11.2)	19 (17.1)	10 (14.9)	39 (23.6)	**<0.001**
6	Inadequate breastfeeding support	112 (16.0)	31 (23.1)	17 (15.3)	13 (19.4)	24 (14.5)	0.647
7	No rooming‐in	74 (10.6)	27 (20.1)	29 (26.1)	33 (49.3)	37 (22.4)	**0.002**
8	Not allowed to stay with the baby as wished	15 (2.1)	2 (1.5)	5 (4.5)	10 (14.9)	5 (3.0)	0.287
9	No exclusive breastfeeding at discharge	123 (17.6)	38 (28.4)	34 (30.6)	25 (37.3)	58 (35.2)	**<0.001**
10	No immediate attention when needed	119 (17.0)	28 (20.9)	28 (25.2)	18 (26.9)	34 (20.6)	**0.014**
Experience of care							
1a	No freedom of movement during labor	51 (7.3)	21 (15.7)	18 (16.2)	‐	‐	**0.001**
1b	No consent requested for vaginal examination in prelabor cesarean	‐	‐	‐	9 (13.4)	11 (6.7)	0.117
2a	No choice of birth position (in SVB)	238 (34.1)	‐	‐	‐	‐	‐
2b	No consent requested (for IVB)	‐	54 (40.3)	‐	‐	‐	‐
2c	No information on newborn after cesarean	‐	‐	9 (8.1)	10 (14.9)	12 (7.3)	**0.032**
3	No clear/effective communication from HCP	107 (15.3)	27 (20.1)	28 (25.2)	18 (26.9)	32 (19.4)	0.057
4	No involvement in choices	132 (18.9)	30 (22.4)	34 (30.6)	23 (34.3)	26 (15.8)	**0.037**
5	Companionship not allowed	235 (33.7)	45 (33.6)	39 (35.1)	29 (43.3)	50 (30.3)	0.636
6	Not treated with dignity	75 (10.7)	14 (10.4)	22 (19.8)	17 (25.4)	17 (10.3)	**0.005**
7	No emotional support	105 (15.0)	36 (26.9)	42 (37.8)	17 (25.4)	29 (17.6)	**<0.001**
8	No privacy	57 (8.2)	14 (10.4)	18 (16.2)	13 (19.4)	14 (8.5)	0.167
9	Abuse (physical/verbal/emotional)	58 (8.3)	18 (13.4)	14 (12.6)	11 (16.4)	12 (7.3)	0.360
10	Informal payment	19 (2.7)	9 (6.7)	4 (3.6)	2 (3.0)	5 (3.0)	0.293
Availability of physical and human resources							
1	No timely care by HCPs at facility arrival	54 (7.7)	11 (8.2)	12 (10.8)	11 (16.4)	4 (2.4)	**0.024**
2	No information on maternal danger signs	231 (33.1)	48 (35.8)	47 (42.3)	24 (35.8)	51 (30.9)	0.261
3	No information on newborn danger signs	284 (40.7)	66 (49.3)	49 (44.1)	26 (38.8)	62 (37.6)	0.435
4	Inadequate room comfort and equipment	8 (1.1)	1 (0.7)	1 (0.9)	1 (1.5)	0 (0.0)	0.871
5	Inadequate number of women per rooms	38 (5.4)	10 (7.5)	4 (3.6)	3 (4.5)	6 (3.6)	0.465
6	Inadequate room cleaning	5 (0.7)	1 (0.7)	1 (0.9)	3 (4.5)	0 (0.0)	0.111
7	Inadequate bathroom	19 (2.7)	1 (0.7)	2 (1.8)	2 (3.0)	0 (0.0)	0.726
8	Inadequate partner visiting hours	169 (24.2)	38 (28.4)	31 (27.9)	14 (20.9)	37 (22.4)	0.371
9	Inadequate HCP number	57 (8.2)	16 (11.9)	18 (16.2)	10 (14.9)	11 (6.7)	**0.022**
10	Inadequate HCP professionalism	7 (1.0)	1 (0.7)	2 (1.8)	3 (4.5)	5 (3.0)	0.256
Reorganizational changes due to COVID‐19							
1	Difficulties in attending routine antenatal visits	133 (19.1)	20 (14.9)	15 (13.5)	21 (31.3)	49 (29.7)	**0.001**
2	Any barriers in accessing the facility	101 (14.5)	14 (10.4)	20 (18.0)	11 (16.4)	37 (22.4)	0.078
3	Inadequate infographics	104 (14.9)	21 (15.7)	21 (18.9)	17 (25.4)	26 (15.8)	0.521
4	Inadequate wards reorganization	155 (22.2)	25 (18.7)	21 (18.9)	24 (35.8)	25 (15.2)	**0.041**
5	Inadequate room reorganization	189 (27.1)	41 (30.6)	32 (28.8)	22 (32.8)	40 (24.2)	0.613
6	Lacking one functioning accessible hand‐washing station	25 (3.6)	4 (3.0)	5 (4.5)	4 (6.0)	4 (2.4)	0.957
7	HCPs not always using PPE	58 (8.3)	10 (7.5)	9 (8.1)	9 (13.4)	14 (8.5)	0.706
8	Insufficient number of HCPs	124 (17.8)	28 (20.9)	26 (23.4)	21 (31.3)	32 (19.4)	0.071
9	Communication inadequate to contain COVID‐19‐related stress	134 (19.2)	26 (19.4)	31 (27.9)	26 (38.8)	38 (23.0)	**0.001**
10	Reduction in QMNC due to COVID‐19	181 (25.9)	34 (25.4)	43 (38.7)	25 (37.3)	45 (27.3)	0.083

Abbreviations: HCP, healthcare professional; IVB, instrumental vaginal birth; PPE, personal protective equipment; QMNC, quality of maternal and newborn care; SVB, spontaneous vaginal birth.

^a^ All the indicators in the domains of provision of care, experience of care, and resources are directly based on WHO standards.

^b^ Indicators with a specified denominator (e.g. 3a, 3b) were tailored to take into account different mode of birth (i.e. spontaneous vaginal, instrumental vaginal, and cesarean). These were calculated on subsamples (e.g. 3a was calculated on spontaneous vaginal births; 3b was calculated on instrumental vaginal births).

^c^ Indicator 6 in the domains of reorganizational changes due to COVID‐19 was defined as: at least one functioning and accessible hand‐washing station (near or inside the room where the mother was hospitalized) supplied with water and soap or with disinfectant alcohol solution.

^d^ *P* values were obtained from the logistic regression model testing quality measures difference by mode of birth adjusted for sociodemographic and obstetric variables (i.e. maternal age, education, year of birth, parity, presence of an obstetrician/gynecologist at birth, multiple birth), language of questionnaire completion, type of hospital, newborn admission to neonatal intensive care or special care baby unit, and mother's admission to intensive care. Bold values are statistically significant.

Further differences were observed within the domain of experience of care. No involvement in choices at birth was reported by 18.9% (*n* = 132) of women with SVB, but by 34.3% (*n* = 23) of those with emergency cesarean before labor (*P* = 0.037). Similarly, 10.7% (*n* = 75) of participants with SVB compared with 25.4% (*n* = 17) of those with emergency cesarean before labor reported that they were not treated with dignity (*P* = 0.005). In addition, 15.0% (*n* = 105) of women with SVB compared with 37.8% (*n* = 42) of women with emergency cesarean during labor indicated no emotional support (*P* < 0.001).

Regarding availability of physical and human resources, significantly more women with emergency cesarean before labor (16.4%, *n* = 11) reported that they did not receive timely care by HCPs at facility arrival compared with others (7.7% [*n* = 54] of women with SVB, *P* = 0.024). Inadequate number of HCPs was reported by 16.2% (*n* = 18) of women with emergency cesarean during labor, 14.9% (*n* = 10) with emergency cesarean before labor, and by 6.7% (*n* = 11) of those with elective cesarean (*P* = 0.022).

In the area of reorganizational changes due to the COVID‐19 pandemic, difficulties in attending routine antenatal visits were reported significantly more often by women with emergency cesarean before labor (31.3%, *n* = 21) than by those with emergency cesarean during labor (13.5%, *n* = 15) (*P* = 0.001). Furthermore, women with emergency cesarean before labor were significantly more likely to indicate that the wards had been inadequately reorganized to reduce the risk of COVID‐19 infection (35.8%, *n* = 24) compared with women with elective cesarean (15.2%, n = 25) (*P* = 0.041). Significantly more women with emergency cesarean before labor (38.8%, *n* = 26) found the communication inadequate to contain COVID‐19‐related stress compared with women with SVB (19.2%, *n* = 134, *P* = 0.001).

### 3.4 QMNC index according to sociodemographic variables, language, and mode of birth

**FIGURE 2:**
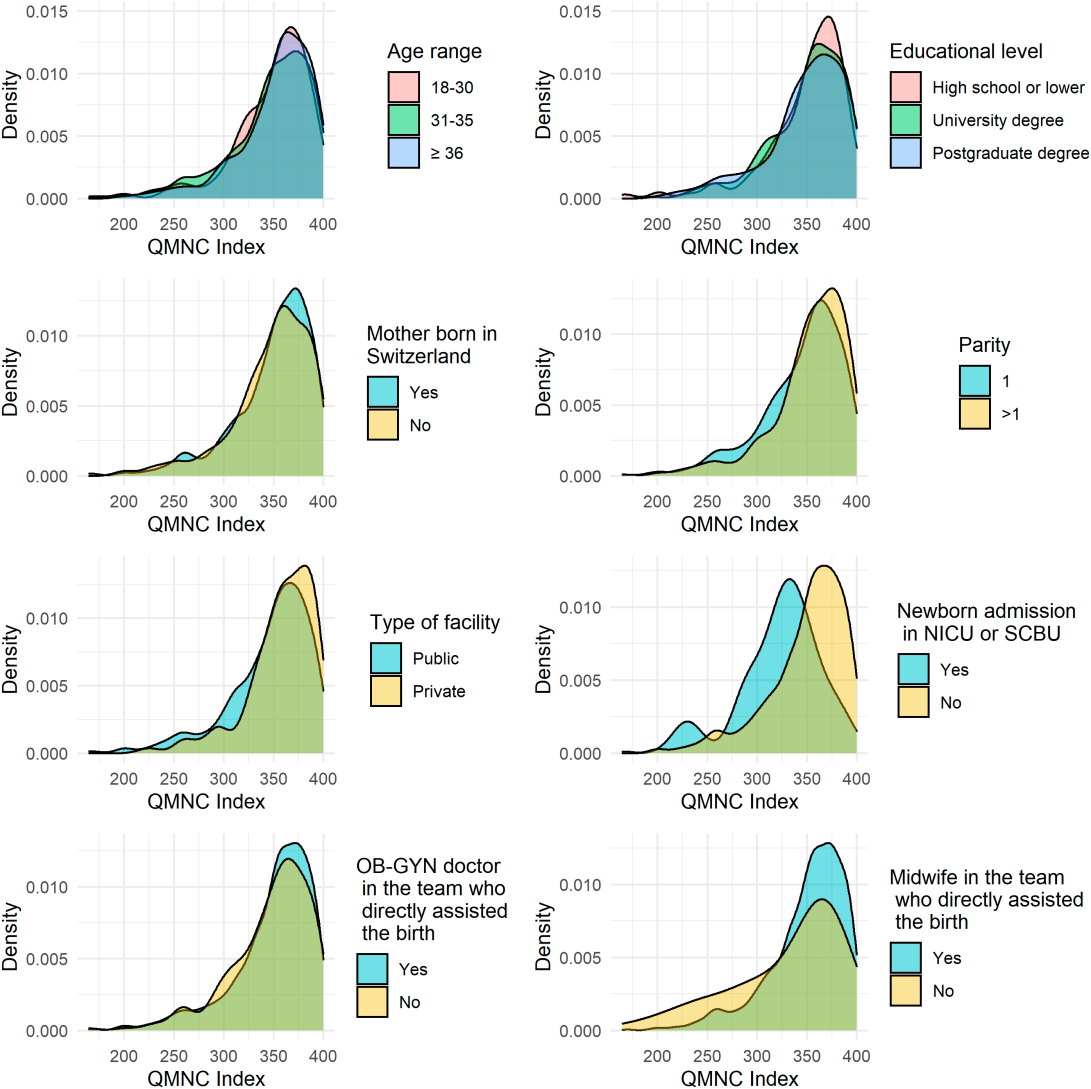
QMNC index by sociodemographic variables. NICU, neonatal intensive care unit; OB‐GYN, obstetrician/gynecologist; QMNC, quality of maternal and newborn care; SCBU, special care baby unit.

**FIGURE 3:**
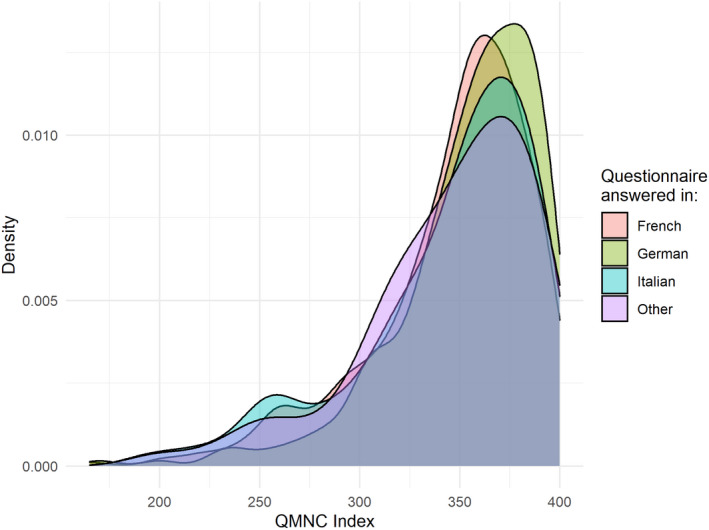
QMNC index by major linguistic region. QMNC, quality of maternal and newborn care.

After adjusting for other variables, multivariate quantile regression showed that a significantly higher QMNC index was found in women aged 36 years and older (coefficient at 0.25th quantile at 11.84, *P* = 0.018) (Figure 2; supporting information Table 3), multiparous women (8.68 at 25th quantile, *P* = 0.043), and births within private settings (25th quantile: 22.11, *P* < 0.001; 50th quantile: 10.00, *P* < 0.001; 75th quantile: 6.25, *P* = 0.01). Women in the German‐speaking region reported a significantly higher QMNC index at the 0.25th, 0.50th, and 0.75th quantiles with increasing coefficients for lower quantiles, 11.84, 7.50, and 7.50, respectively (Figure 3; supporting information Table 3).

**FIGURE 4:**
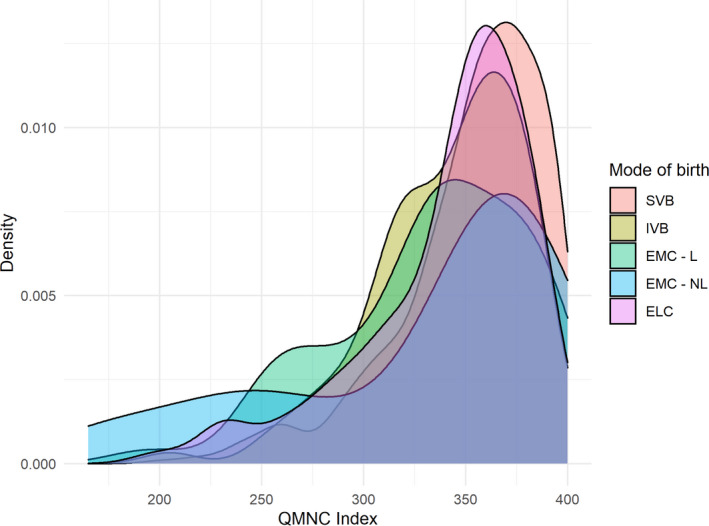
QMNC index by mode of birth. ELC, planned or elective cesarean before labor; EMC—L, emergency cesarean during labor; EMC—NL, emergency cesarean before labor; IVB, instrumental vaginal birth; QMNC, quality of maternal and newborn care; SVB, spontaneous vaginal birth.

Women who underwent IVB (coefficient at 0.50th quantile at −12.50, *P* = 0.003) or elective cesarean (coefficient at 0.50th and 0.75th quantile at −10.00, *P* = 0.007 and −11.25, *P* = 0.005, respectively) or emergency cesarean during labor (coefficient at 0.50th quantile at −22.50, *P* < 0.001) reported a significantly lower median QMNC index than women with SVB (Figure 4).

## 4 DISCUSSION

To our knowledge, this is the first study in Switzerland investigating QMNC as perceived by women, and comparing results by linguistic regions and mode of birth during the COVID 19 pandemic. More than a quarter of women reported limitations of QMNC due to the COVID‐19 pandemic. Although results for several quality measures indicate high QMNC in Switzerland, some findings suggested preventable gaps in QMNC. Many quality measures significantly differed by linguistic region and mode of birth. The highest QMNC index was reported in the German‐speaking region. Women in the French‐speaking region were most likely to report lack of HCPs, lack of clear communication, and lack of information on maternal complications that may have left them feeling uninvolved in medical decisions. Women responding in other than national languages identified difficulties mainly linked to a lack of information on newborn complications, and deficient communication with HCPs, particularly to reduce their stress related to COVID‐19, and inadequate infographics. Concerning mode of birth, participants who had undergone an emergency cesarean when not in labor expressed numerous poor‐quality items in comparison with the other modes of birth (e.g. lack of involvement in choices at birth, difficulties in attending routine antenatal visits, inadequate ward reorganization). They also reported shortcomings linked to not receiving timely care by HCPs at arrival along with scarce HCP numbers.

Data highlighting poor quality measures during the pandemic are consistent with other studies in Switzerland showing that almost 40% of women reported a negative impact of COVID‐19 on their pregnancy and breastfeeding experiences (e.g. concerns about restrictive measures, anxiety during social contact, additional stress).^26^ The present study contributed to new knowledge on pregnancy experience during COVID‐19 as, to the best of our knowledge, no comparisons across Swiss linguistic regions have been investigated.

Observed differences in the QMNC scores across linguistic regions can be explained by multiple factors. In the German‐speaking region of Switzerland, lower numbers of new cases of COVID‐19 and higher interpersonal trust levels (i.e. belief that fellow citizens will respect the rules of distancing and individual responsibility) have been reported compared with the other regions.^27^ These may be reasons that pregnant women perceived a lower impact of the COVID‐19 pandemic on QMNC. In contrast, the Italian‐ and French‐speaking regions experienced more COVID‐19 cases,^27^ which could explain why women in these regions perceived an inadequate number of HCPs, and that HCPs were unable to provide clear and effective communication. Previous studies observed that conflicting and constantly changing information from HCPs reduced the perceived quality of care provided.^28^ A shortage of HCPs has been observed for several years in Switzerland, and during the pandemic numbers did seem insufficient to guarantee adequate assistance. According to the Swiss Health Observatory,^29^ the need to provide replacement forces of HCPs remains enormous for the coming decade. Finally, a high proportion of women responding in languages other than the national languages reported inadequacy in communication to contain COVID‐19‐related stress (e.g. posters, images). During the COVID‐19 pandemic, women and their families need to be adequately informed and supported in perinatal care.^30^ Possible additional explanations include a range of health system issues such as competencies of HCPs, type of infrastructure, organization of care, and budget allocation, which possibly varied in different regions considering the autonomy of decision found in the Swiss cantons.

Observed differences in QMNC scores by mode of birth are consistent with previous studies from the IMAgiNE EURO project,^18^, ^31^, ^32^, ^33^, ^34^, ^35^ and with previous research in Switzerland showing that women with an emergency cesarean have the highest risks of an adverse birth experience.^36^ Literature shows that a divergence between the mode of delivery planned by the women and the one they achieved may be one of the factors associated with a negative birth experience.^37^ In the present study, women with SVB and elective cesarean scored higher on the QMNC index, especially in the provision of care items (e.g. early and exclusive breastfeeding at discharge) and in the experience of care items (e.g. involvement in choices at birth, treated with dignity, emotional support). This study highlights that a negative birth experience cannot be attributed to birth mode alone, but also to divergence between planned and actual delivery. Both factors might lead to poor quality measures.

Results from systematic reviews indicating that the COVID‐19 pandemic increased the rate of cesarean were not reflected in the results of the present study; on the contrary, the rate of cesarean birth was slightly lower compared with the national average in 2017.^38^

The finding that multiparous older women and those giving birth in private facilities had a significantly higher QMNC index compared with younger and primiparous women and women who gave birth in public facilities confirms previous findings.^39^, ^40^ However, women giving birth in private facilities typically actively chose this option, and this per se may bias the perception of care in the service they expressed preference for. Nevertheless, autonomy and pain relief satisfaction were reported more often in women giving birth in private facilities than those in public facilities.^41^ This seems to be a possible explanation for the higher QMNC scores for women giving birth in private facilities. Additionally, women giving birth in private facilities differ from those giving birth in public facilities, usually higher social status and fewer comorbidities,^42^ and this may also explain a higher QMNC. In our questionnaire we did not have details on women's comorbidities and complications, thus we lacked information to better compare groups.

One of the strengths of this study was the use of a validated standardized questionnaire, including a set of 40 prioritized WHO standards‐based quality measures, available in 24 languages.^18^ Many of the characteristics of the sample were similar to the latest national statistical reports (e.g. IVB, cesarean, emergency cesarean, or type of facility).^38^ Nevertheless, the study sample is not representative of the Swiss population as some characteristics differed from its general description. Despite a majority of German speakers in Switzerland,^43^ in this study more women responded in French; this is certainly due to the recruitment period, which started later in the German part of Switzerland. Therefore, this under‐representation of the German‐speaking population potentially impacted the results. This sample seems to include a majority of highly educated women, however the categories used for the educational level in the questionnaire were not fully aligned with those used in national reports, thus making a comparison difficult. In addition, we cannot guarantee that all women answering in a national language lived in the corresponding linguistic region. Moreover, a potential positive selection bias toward women with a higher interest in participating cannot be excluded. Finally, no QMNC measurements prior to the COVID‐19 pandemic have been recorded in Switzerland, which is a limitation for discussion of the impact of the pandemic. Further studies should explore the QMNC over time.

In conclusion, to ensure and safeguard the standards of QMNC^1^ even during unexpected events such as pandemics, national or regional guidelines need to take into account women's experience and the perceived QMNC. Limitations to QMNC were reported by more than a quarter of women because of the pandemic. Some women felt there was an insufficient number of HCPs to guarantee adequate assistance during the COVID‐19 pandemic. Moreover, women perceived not receiving sufficient information on their own health and that of their newborn, which is particularly alarming. Policy makers and hospitals managers should be aware that providing sufficient and adequate human resources is likely to be the first element to secure during acute events such as a pandemic. Additionally, HCPs should ensure that effective communication and respect for women's dignity are provided, as they appear to be deprioritized during emergency situations, especially reported by women who did not respond in one of the national languages. Finally, young age, primiparity, emergency cesarean not in labor, or IVB should be considered by HCPs as warning signs as they may lead to poorer quality of care measures. These recommendations may help similar settings improve some of the key quality measures of QMNC that are particularly impacted during pandemics.

## AUTHOR CONTRIBUTIONS

Marzia Lazzerini conceived the IMAgiNE EURO study, with major inputs from Emanuelle Pessa Valente, Benedetta Covi, Ilaria Mariani, and additional input from all other authors. All authors promoted the surveys and supported the process of data collection. Claire de Labrusse, Alessia Abderhalden‐Zellweger, Anouck Pfund, Michael Gemperle, Susanne Grylka‐Baeschlin, and Antonia N. Mueller conceived the present article, with major inputs from Marzia Lazzerini. Ilaria Mariani analyzed the data, with major inputs from Claire de Labrusse, Alessia Abderhalden‐Zellweger, Anouck Pfund, Michael Gemperle, Susanne Grylka‐Baeschlin, and Antonia N. Mueller. All authors approved the final version of the manuscript for submission.

## CONFLICT OF INTEREST

The authors have no conflicts of interest to declare.

## IMAgiNE EURO study group

**Bosnia and Herzegovina:** Amira Ćerimagić, NGO Baby Steps, Sarajevo; **Croatia:** Daniela Drandić, Roda – Parents in Action, Zagreb; Magdalena Kurbanović, Faculty of Health Studies, University of Rijeka, Rijeka; **France:** Rozée Virginie, Elise de La Rochebrochard, Sexual and Reproductive Health and Rights Research Unit, Institut National d’Études Démographiques (INED), Paris; Kristina Löfgren, Baby‐friendly Hospital Initiative (IHAB); **Germany:** Céline Miani, Stephanie Batram‐Zantvoort, Lisa Wandschneider, Department of Epidemiology and International Public Health, School of Public Health, Bielefeld University, Bielefeld; **Italy:** Marzia Lazzerini, Emanuelle Pessa Valente, Benedetta Covi, Ilaria Mariani, Institute for Maternal and Child Health IRCCS “Burlo Garofolo”, Trieste; Sandra Morano, Medical School and Midwifery School, Genoa University, Genoa; **Israel:** Ilana Chertok, Ohio University, School of Nursing, Athens, Ohio, USA and Ruppin Academic Center, Department of Nursing, Emek Hefer; Rada Artzi‐Medvedik, Department of Nursing, The Recanati School for Community Health Professions, Faculty of Health Sciences at Ben‐Gurion University (BGU) of the Negev; **Latvia:** Elizabete Pumpure, Dace Rezeberga, Gita Jansone‐Šantare, Department of Obstetrics and Gynecology, Riga Stradins University and Riga Maternity Hospital, Riga; Dārta Jakovicka, Faculty of Medicine, Riga Stradins University, Rīga; Agnija Vaska, Riga Maternity Hospital, Riga; Anna Regīna Knoka, Faculty of Medicine, Riga Stradins University, Rīga; Katrīna Paula Vilcāne, Faculty of Public Health and Social Welfare, Riga Stradins University, Riga; **Lithuania:** Alina Liepinaitienė, Andželika Kondrakova, Kaunas University of Applied Sciences, Kaunas; Marija Mizgaitienė, Simona Juciūtė, Kaunas Hospital of the Lithuanian University of Health Sciences, Kaunas; **Luxembourg:** Maryse Arendt, Professional Association of Lactation Consultants in Luxembourg; Barbara Tasch, Professional Association of Lactation Consultants in Luxembourg and Neonatal Intensive Care Unit, KannerKlinik, Centre Hospitalier de Luxembourg, Luxembourg; **Norway:** Ingvild Hersoug Nedberg, Sigrun Kongslien, Department of Community Medicine, UiT The Arctic University of Norway, Tromsø; Eline Skirnisdottir Vik, Department of Health and Caring Sciences, Western Norway University of Applied Sciences, Bergen; **Poland:** Barbara Baranowska, Urszula Tataj‐Puzyna, Maria Węgrzynowska, Department of Midwifery, Centre of Postgraduate Medical Education, Warsaw; **Portugal:** Raquel Costa, EPIUnit ‐ Instituto de Saúde Pública, Universidade do Porto, Porto; Laboratório para a Investigação Integrativa e Translacional em Saúde Populacional, Porto; Lusófona University/HEI‐Lab: Digital Human‐environment Interaction Labs, Lisbon; Catarina Barata, Instituto de Ciências Sociais, Universidade de Lisboa; Teresa Santos, Universidade Europeia, Lisboa and Plataforma CatólicaMed/Centro de Investigação Interdisciplinar em Saúde (CIIS) da Universidade Católica Portuguesa, Lisbon; Carina Rodrigues, EPIUnit ‐ Instituto de Saúde Pública, Universidade do Porto, Porto and Laboratório para a Investigação Integrativa e Translacional em Saúde Populacional, Porto; Heloísa Dias, Regional Health Administration of the Algarve; **Romania:** Marina Ruxandra Otelea, University of Medicine and Pharmacy Carol Davila, Bucharest and SAMAS Association, Bucharest; **Serbia:** Jelena Radetić, Jovana Ružičić, Centar za mame, Belgrade; **Slovenia:** Zalka Drglin, Barbara Mihevc Ponikvar, Anja Bohinec, National Institute of Public Health, Ljubljana; **Spain:** Serena Brigidi, Department of Anthropology, Philosophy and Social Work, Medical Anthropology Research Center (MARC), Rovira i Virgili University (URV), Tarragona; Lara Martín Castañeda, Institut Català de la Salut, Generalitat de Catalunya; **Sweden:** Helen Elden, Verena Sengpiel, Institute of Health and Care Sciences, Sahlgrenska Academy, University of Gothenburg and Department of Obstetrics and Gynecology, Region Västra Götaland, Sahlgrenska University Hospital, Gothenburg; Karolina Linden, Institute of Health and Care Sciences, Sahlgrenska Academy, University of Gothenburg; Mehreen Zaigham, Department of Obstetrics and Gynecology, Institution of Clinical Sciences Lund, Lund University, Lund and Skåne University Hospital, Malmö; **Switzerland:** Claire de Labrusse, Alessia Abderhalden, Anouck Pfund, Harriet Thorn, School of Health Sciences (HESAV), HES‐SO University of Applied Sciences and Arts Western Switzerland, Lausanne; Susanne Grylka‐Baeschlin, Michael Gemperle, Antonia N. Mueller, Research Institute of Midwifery, School of Health Sciences, ZHAW Zurich University of Applied Sciences, Winthertur.

## DISCLAIMER

The authors alone are responsible for the views expressed in this article and they do not necessarily represent the views, decisions, or policies of the institutions with which they are affiliated.

## 5 Ethical aspects

The study protocol was approved by the Institutional Review Board of the IRCCS “Burlo Garofolo”, Trieste, Italy (IRB‐BURLO 05/2020 15.07.2020). Because it was a voluntary anonymous survey on mother's views on QMNC, this study did not fall under the Human Research Act (art. 2) according to the Ethics Committee of the Canton of Vaud and therefore did not require any further ethical approval in Switzerland (CER‐VD, information on July 9, 2021). Data were transmitted, stored, encrypted, and analyzed in Italy. When accessing the link and prior to participation, women were informed about the aims and methods of the study, including their rights to refuse to participate or to withdraw at any time. Informed consent before answering the questionnaire was obtained.

## Supporting information


Table S1
Table S2
Table S3

## Data Availability

Data can be made available on reasonable request to the corresponding author.

## References

[1] World Health Organization . Standards for Improving Quality of Maternal and Newborn Care in Health Facilities. WHO; 2016.

[2] Tunçalp Ӧ , Were WM , MacLennan C , et al. Quality of care for pregnant women and newborns‐the WHO vision. BJOG. 2015;122:1045‐1049. 25929823 10.1111/1471-0528.13451 PMC5029576

[3] International Confederation of Midwives . Women's rights in childbirth must be upheld during the coronavirus pandemic. 2021.

[4] World Health Organization . COVID‐19: Operational Guidance for Maintaining Essential Health Services During an Outbreak. WHO; 2020.

[5] de Labrusse C , Ramelet AS , Humphrey T , Maclennan SJ . Patient‐centered care in maternity services: a critical appraisal and synthesis of the literature. Womens Health Issues. 2016;26:100‐109. 26549243 10.1016/j.whi.2015.09.003

[6] Chmielewska B , Barratt I , Townsend R , et al. Effects of the COVID‐19 pandemic on maternal and perinatal outcomes: a systematic review and meta‐analysis. Lancet Glob Health. 2021;9:e759‐e772. 33811827 10.1016/S2214-109X(21)00079-6 PMC8012052

[7] Gurol‐Urganci I , Jardine JE , Carroll F , et al. Maternal and perinatal outcomes of pregnant women with SARS‐CoV‐2 infection at the time of birth in England: national cohort study. Am J Obstet Gynecol. 2021;225:522.e1‐522.e11. 10.1016/j.ajog.2021.05.016 PMC8135190 34023315

[8] Montagnoli C , Zanconato G , Ruggeri S , Cinelli G , Tozzi AE . Restructuring maternal services during the covid‐19 pandemic: early results of a scoping review for non‐infected women. Midwifery. 2021;94:102916. 33412360 10.1016/j.midw.2020.102916 PMC7832106

[9] Dell'Utri C , Manzoni E , Cipriani S , et al. Effects of SARS Cov‐2 epidemic on the obstetrical and gynecological emergency service accesses. What happened and what shall we expect now? Eur J Obstet Gynecol Reprod Biol. 2020;254:64‐68. 32942077 10.1016/j.ejogrb.2020.09.006 PMC7476452

[10] Linden K , Domgren N , Zaigham M , Sengpiel V , Andersson ME , Wessberg A . Being in the shadow of the unknown ‐ Swedish women's lived experiences of pregnancy during the COVID‐19 pandemic, a phenomenological study. Women Birth. 2022;35:440‐446. 34602340 10.1016/j.wombi.2021.09.007 PMC9364685

[11] Meaney S , Leitao S , Olander EK , Pope J , Matvienko‐Sikar K . The impact of COVID‐19 on pregnant womens' experiences and perceptions of antenatal maternity care, social support, and stress‐reduction strategies. Women Birth. 2022;35:307‐316. 33994134 10.1016/j.wombi.2021.04.013 PMC9051126

[12] Naurin E , Markstedt E , Stolle D , et al. Pregnant under the pressure of a pandemic: a large‐scale longitudinal survey before and during the COVID‐19 outbreak. Eur J Public Health. 2021;31:7‐13. 10.1093/eurpub/ckaa223 PMC7717243 33231625

[13] Desson Z , Lambertz L , Peters JW , Falkenbach M , Kauer L . Europe's Covid‐19 outliers: German, Austrian and Swiss policy responses during the early stages of the 2020 pandemic. Health Policy Technol. 2020;9:405‐418. 33520639 10.1016/j.hlpt.2020.09.003 PMC7834269

[14] Federal Office of Public Health FOPH . COVID‐19 Switzerland. Information on the current situation, as of 7 June 2022. Accessed June 7, 2022. https://www.covid19.admin.ch/en/epidemiologic/case?demoView=graph&time=total

[15] Surbek D , Baud D . Lettre d'experts SSGO gynécologie suisse: infection à coronavirus COVID‐19. August 8, 2020, Société Suisse de Gynécologie et d'Obstétrique SSGO. Accessed August 29, 2022. https://www.sggg.ch/fileadmin/user_upload/Dokumente/1_Ueber_uns/Empfehlung_Coronavirusinfektion_COVID‐19_05.08.2020_FR.pdf

[16] Swiss Federation of Midwives . Prise de position de la Fédération suisse des sages‐femmes pour l'accouchement pendant la pandémie COVID‐19. November 4, 2020. Accessed August 29, 2022. https://www.hebamme.ch/wp‐content/uploads/2020/11/Prise‐de‐position‐de‐la‐Federation‐suisse‐des‐sages‐femmes‐pour‐laccouchement‐pendant‐la‐pandemie‐COVID‐19.pdf

[17] IMAgiNE EURO Improving Maternal Newborn Care in the European Region. Accessed June 22, 2022. https://www.burlo.trieste.it/ricerca/imagine‐euro‐improving‐maternal‐newborn‐care‐euro‐region

[18] Lazzerini M , Covi B , Mariani I , et al. Quality of facility‐based maternal and newborn care around the time of childbirth during the COVID‐19 pandemic: online survey investigating maternal perspectives in 12 countries of the WHO European Region. Lancet Reg Health Eur. 2022;13:100268. 34977838 10.1016/j.lanepe.2021.100268 PMC8703114

[19] von Elm E , Altman DG , Egger M , et al. The Strengthening the Reporting of Observational Studies in Epidemiology (STROBE) Statement: guidelines for reporting observational studies. Int J Surg. 2014;12:1495‐1499. 25046131 10.1016/j.ijsu.2014.07.013

[20] Federal Statistical Office . Langues principales selon les cantons. 2020. February 2022. Accessed August 29, 2022. https://www.bfs.admin.ch/bfs/en/home/statistics/catalogues‐databases/tables.assetdetail.21344039.html

[21] De Pietro C , Camenzind P , Sturny I , et al. Switzerland: health system review. Health Syst Transit. 2015;17:1‐288. xix. 26766626

[22] National Institute for Health and Care Excellence. Intrapartum carefor healthy women and babies. Clinical guideline [CG190] , 2017. Accessed February 9, 2022. https://www.nice.org.uk/guidance/cg190 31820894

[23] Semaan A , Audet C , Huysmans E , et al. Voices from the frontline: findings from a thematic analysis of a rapid online global survey of maternal and newborn health professionals facing the COVID‐19 pandemic. BMJ Glob Health. 2020;5:e002967. 10.1136/bmjgh-2020-002967 PMC7335688 32586891

[24] Koenker R . Quantile Regression. Cambridge University Press; 2005.

[25] R Core Team . R: A Language and Environment for Statistical Computing. R Foundation for Statistical Computing. 2020. https://www.eea.europa.eu/data‐and‐maps/indicators/oxygen‐consuming‐substances‐in‐rivers/r‐development‐core‐team‐2006

[26] Lambelet V , Ceulemans M , Nordeng H , et al. Impact of the COVID‐19 pandemic on Swiss pregnant and breastfeeding women ‐ a cross‐sectional study covering the first pandemic wave. Swiss Med Wkly. 2021;151:w30009. 34546015 10.4414/smw.2021.w30009

[27] Deopa N , Fortunato P . Coronagraben in Switzerland: culture and social distancing in times of COVID‐19. J Popul Econ. 2021;34:1355‐1383. 34334956 10.1007/s00148-021-00865-y PMC8315728

[28] Vasilevski V , Sweet L , Bradfield Z , et al. Receiving maternity care during the COVID‐19 pandemic: experiences of women's partners and support persons. Women Birth. 2022;35:298‐306. 33941497 10.1016/j.wombi.2021.04.012 PMC8075817

[29] Merçay C , Grünig A , Dolder P . Personnel de santé en Suisse – Rapport national 2021. Effectifs, besoins, offre et mesures pour assurer la relève. Swiss Health Observatory (OBSAN); 2021: 118.

[30] Homer C . Our words matter–now more than ever. O&G Magazine 2021. 23(4). Accessed August 29, 2022. https://www.ogmagazine.org.au/23/4‐23/our‐words‐matter‐now‐more‐than‐ever/

[31] Zaigham M , Linden K , Sengpiel V , et al. Large gaps in the quality of healthcare experienced by Swedish mothers during the COVID‐19 pandemic: a cross‐sectional study based on WHO standards. Women Birth. 2022;35:619‐627. 35123922 10.1016/j.wombi.2022.01.007 PMC8784577

[32] Drandić D , Drglin Z , Mihevc Ponkivar B , et al. Women’s perspectives on the quality of hospital maternal and newborn care around the time of childbirth during the COVID‐19 pandemic: results from the IMAgiNE EURO study in Slovenia, Croatia, Serbia, and Bosnia‐Herzegovina. Int J Gynecol Obstet. 2022;159(Suppl 1):54‐69. 10.1002/ijgo.14457 PMC9877897 36530003

[33] Pumpure E , Jakovicka D , Mariani I , et al. Women’s perspectives on the quality of maternal and newborn care in childbirth during the COVID‐19 pandemic in Latvia: results from the IMAgiNE EURO study on 40 WHO standards‐based quality measures. Int J Gynecol Obstet. 2022;159(Suppl 1):97‐112. 10.1002/ijgo.14461 PMC9878132 36530013

[34] Otelea MR , Simionescu AA , Mariani I , et al. Women’s assessment of the quality of hospital‐based perinatal care by mode of birth in Romania during the COVID‐19 pandemic: Results from the IMAgiNE EURO study. Int J Gynecol Obstet. 2022;159(Supp 1):126‐136. 10.1002/ijgo.14482 PMC9877960 36530009

[35] Hersoug Nedberg I , Skirnisdottir Vik E , Kongslien S , et al. Quality of health care around the time of childbirth during the COVID‐19 pandemic: Results from the IMAgiNE EURO study in Norway and trends over time. Int J Gynecol Obstet. 2022;159(Supp 1):85‐96. 10.1002/ijgo.14460 PMC9877678 36530008

[36] Carquillat P , Boulvain M , Guittier MJ . How does delivery method influence factors that contribute to women's childbirth experiences? Midwifery. 2016;43:21‐28. 27825057 10.1016/j.midw.2016.10.002

[37] Blomquist JL , Quiroz LH , Macmillan D , McCullough A , Handa VL . Mothers' satisfaction with planned vaginal and planned cesarean birth. Am J Perinatol. 2011;28:383‐388. 21380993 10.1055/s-0031-1274508 PMC3086342

[38] Federal Statistical Office . Statistique médicale des hôpitaux. Accouchements et santé maternelle en 2017. May 2019. Accessed August 29, 2022. https://www.bfs.admin.ch/bfs/fr/home/statistiques/sante/etat‐sante/reproductive.assetdetail.8369419.html

[39] Euro‐Peristat Project . European Perinatal Health Report. Core indicators of the health and care of pregnant women and babies in Europe in 2015. November 2018.

[40] Weeks F , Pantoja L , Ortiz J , Foster J , Cavada G , Binfa L . Labor and birth care satisfaction associated with medical interventions and accompaniment during labor among Chilean women. J Midwifery Womens Health. 2017;62:196‐203. 27543442 10.1111/jmwh.12499

[41] Moran PS , Daly D , Wuytack F , et al. Predictors of choice of public and private maternity care among nulliparous women in Ireland, and implications for maternity care and birth experience. Health Policy. 2020;124:556‐562. 32284156 10.1016/j.healthpol.2020.02.008

[42] Lazzerini M , Pessa Valente E , Covi B , et al. Rates of instrumental vaginal birth and cesarean delivery and quality of maternal and newborn health care in private versus public facilities: results of the IMAgiNE EURO study in 16 countries. Int J Gynecol Obstet. 2022; 159(Supp 1):22‐38. 10.1002/ijgo.14458 PMC10108180 36530007

[43] Federal Statistical Office . Languages declared as main languages. 2022. Accessed August 29, 2022. https://www.bfs.admin.ch/bfs/en/home/statistics/population/languages‐religions.assetdetail.21344061.html

