# Supplementary material for: Quality of maternal and newborn care in Switzerland during the COVID‐19 pandemic: A cross‐sectional study based on WHO quality standards
Source: Int J Gynaecol Obstet. 2022 Dec 18;159(Suppl 1):70–84. doi: 10.1002/ijgo.14456 (PMC9877813; doi:10.1002/ijgo.14456)
Supplement: Supplementary file 1 — Table S1 Table S2 Table S3 [file IJGO-159-70-s001.docx]

**Supporting information** **Table 1**. List of the 40 quality measures for each WHO domain of care^a^

| Provision of care | Experience of care | Availability of physical and human resources | Reorganizational changes due to COVID-19 |
| --- | --- | --- | --- |
| 1. No pain relief during labor 2. Mode of birth 3. 3a. Episiotomy (in SVB), 3b. Fundal pressure (in IVB), 3c. No pain relief after cesarean 4. No skin to skin 5. No early breastfeeding 6. Inadequate breastfeeding support 7. No rooming-in 8. Not allowed to stay with the baby as wished 9. No exclusive breastfeeding at discharge 10. No immediate attention when needed | 1. 1a. No freedom of movements during labor, 1b. No consent requested for vaginal examination in prelabor cesarean 2. 2a. No choice of birth position (in SVB), 2b. No consent requested (for IVB), 2c. No information on newborn (in cesarean) 3. No clear/effective communication from HCP 4. No involvement in choices 5. Companionship not allowed 6. Not treated with dignity 7. No emotional support 8. No privacy 9. Abuse (physical/verbal/emotional) 10. Informal payment | 1. No timely care by HCP at facility arrival 2. No information on maternal danger signs 3. No information on newborn danger signs 4. Inadequate room comfort and equipment 5. Unacceptable number of women per rooms 6. Inadequate room cleaning 7. Inadequate bathroom 8. Inadequate partner visiting hours 9. Inadequate HCP number 10. Inadequate HCP professionalism | 1. Difficulties in attending routine antenatal visits 2. Any barriers in accessing the facility 3. Inadequate info graphics 4. Inadequate wards reorganization 5. Inadequate room reorganization 6. Lacking one functioning accessible hand-washing station 7. HCP not always using PPE 8. Insufficient HCP number 9. Communication inadequate to contain COVID-19-related stress 10. Reduction in QMNC due to COVID-19 |

Abbreviations: HCP, healthcare professional; IVB, instrumental vaginal birth; PPE, personal protective equipment; QMNC, quality of maternal and newborn care; SVB, spontaneous vaginal birth.

^a^ All the indicators in the domains of provision of care, experience of care, and resources are directly based on WHO standards.

^b^ Indicators identified with letters (e.g. 3a, 3b) were tailored to take into account different mode of birth (i.e. spontaneous vaginal, instrumental vaginal, and cesarean). These were calculated on subsamples (e.g. 3a was calculated on spontaneous vaginal births; 3b was calculated on instrumental vaginal births).

^c^ Indicator 6 in the domain of reorganizational changes due to COVID-19 was defined as: at least one functioning and accessible hand-washing station (near or inside the room where the mother was hospitalized) supplied with water and soap or with disinfectant alcohol solution.

**Supporting information Table 2**. Quality of maternal and newborn care (QMNC) index score system

|  | Number of questions | Score options for each answer | Total score (range) |
| --- | --- | --- | --- |
| Provision of care | 10 | 0, 5, 10 | 0–100 |
| Experience of care | 10 | 0, 5, 10 | 0–100 |
| Human and physical resources | 10 | 0, 5, 10 | 0–100 |
| COVID-19 | 10 | 0, 5, 10 | 0–100 |
| QMNC index | 40 | 0, 5, 10 | 0–400 |

**Supporting information Table 3.** Multivariable quantile regression estimates (n=1175)

|  | 0.25th centile | | | | | | 0.50th centile | | | | | | 0.75th centile | | | | | |
| --- | --- | --- | --- | --- | --- | --- | --- | --- | --- | --- | --- | --- | --- | --- | --- | --- | --- | --- |
|  |  | | 95% CI | |  | |  | | 95% CI | |  | |  | 95% CI | | |  | |
|  | Coefficient | Lower limit | | Upper limit | | *P* value | Coefficient | Lower limit | | Upper limit | | *P* value | Coefficient | | Lower limit | Upper limit | | *P* value |
| **Language** |  |  | |  | |  |  |  | |  | |  |  | |  |  | |  |
| French (reference) |  |  | |  | |  |  |  | |  | |  |  | |  |  | |  |
| German | 11.84 | 3.13 | | 20.55 | | 0.008 | 7.50 | 2.04 | | 12.96 | | 0.007 | 7.50 | | 2.61 | 12.39 | | 0.003 |
| Italian | -6.58 | -20.76 | | 7.61 | | 0.363 | 2.50 | -6.98 | | 11.98 | | 0.605 | 5.00 | | -4.33 | 14.33 | | 0.293 |
| Other | -12.11 | -25.71 | | 1.50 | | 0.081 | 2.50 | -11.87 | | 16.87 | | 0.733 | 2.50 | | -8.87 | 13.87 | | 0.666 |
| **Mode of birth** |  |  | |  | |  |  |  | |  | |  |  | |  |  | |  |
| SVB^a^ (reference) |  |  | |  | |  |  |  | |  | |  |  | |  |  | |  |
| IVB | -9.21 | -21.56 | | 3.14 | | 0.144 | -12.50 | -20.74 | | -4.26 | | 0.003 | -7.50 | | -16.14 | 1.14 | | 0.089 |
| EC-L | -34.47 | -76.39 | | 7.44 | | 0.107 | -22.50 | -35.02 | | -9.98 | | <0.001 | -10.00 | | -28.23 | 8.23 | | 0.282 |
| EC-NL | -64.74 | -148.84 | | 19.36 | | 0.131 | 0.00 | -15.28 | | 15.28 | | >0.99 | -5.00 | | -14.00 | 4.00 | | 0.276 |
| ELC | -13.95 | -31.68 | | 3.79 | | 0.123 | -10.00 | -17.26 | | -2.74 | | 0.007 | -11.25 | | -19.05 | -3.45 | | 0.005 |
| **Age range, y** |  |  | |  | |  |  |  | |  | |  |  | |  |  | |  |
| 18–30 | 8.68 | -0.53 | | 17.89 | | 0.065 | 2.50 | -4.24 | | 9.24 | | 0.467 | -2.50 | | -8.50 | 3.50 | | 0.414 |
| 31–35 (reference) |  |  | |  | |  |  |  | |  | |  |  | |  |  | |  |
| ≥36 | 11.84 | 2.06 | | 21.62 | | 0.018 | 2.50 | -3.41 | | 8.41 | | 0.406 | 1.25 | | -3.94 | 6.44 | | 0.637 |
| **Educational level** |  |  | |  | |  |  |  | |  | |  |  | |  |  | |  |
| High school or lower | 1.84 | -7.99 | | 11.67 | | 0.713 | 0.00 | -6.90 | | 6.90 | | >0.99 | -3.75 | | -9.68 | 2.18 | | 0.214 |
| University or higher | 4.47 | -5.38 | | 14.33 | | 0.373 | 0.00 | -5.72 | | 5.72 | | >0.99 | 0.00 | | -6.43 | 6.43 | | >0.99 |
| Postgraduate degree/Master/Doctorate or higher (reference) |  |  | |  | |  |  |  | |  | |  |  | |  |  | |  |
| **Birth after June 2021** |  |  | |  | |  |  |  | |  | |  |  | |  |  | |  |
| No (reference) |  |  | |  | |  |  |  | |  | |  |  | |  |  | |  |
| Yes | 8.42 | -0.14 | | 16.98 | | 0.054 | 5.00 | -0.55 | | 10.55 | | 0.077 | 5.00 | | -0.67 | 10.67 | | 0.084 |
| **Mother born in Switzerland** |  |  | |  | |  |  |  | |  | |  |  | |  |  | |  |
| Yes (reference) |  |  | |  | |  |  |  | |  | |  |  | |  |  | |  |
| No | 5.53 | -3.31 | | 14.36 | | 0.22 | 0.00 | -6.99 | | 6.99 | | >0.99 | 2.50 | | -4.43 | 9.43 | | 0.479 |
| **Type of facility** |  |  | |  | |  |  |  | |  | |  |  | |  |  | |  |
| Public (reference) |  |  | |  | |  |  |  | |  | |  |  | |  |  | |  |
| Private | 22.11 | 14.31 | | 29.90 | | <0.001 | 10.00 | 4.86 | | 15.14 | | <0.001 | 6.25 | | 1.51 | 10.99 | | 0.01 |
| **Parity** |  |  | |  | |  |  |  | |  | |  |  | |  |  | |  |
| 1 (reference) |  |  | |  | |  |  |  | |  | |  |  | |  |  | |  |
| >1 | 8.68 | 0.27 | | 17.09 | | 0.043 | 5.00 | -0.71 | | 10.71 | | 0.086 | 5.00 | | -0.31 | 10.31 | | 0.065 |
| **OB/GYN doctor in the team who assisted the birth** |  |  | |  | |  |  |  | |  | |  |  | |  |  | |  |
| No (reference) |  |  | |  | |  |  |  | |  | |  |  | |  |  | |  |
| Yes | 5.26 | -3.19 | | 13.71 | | 0.222 | 5.00 | -0.82 | | 10.82 | | 0.092 | 2.50 | | -2.53 | 7.53 | | 0.329 |
| Intercept | 309.21 | 296.40 | | 322.02 | | <0.001 | 347.50 | 338.66 | | 356.34 | | <0.001 | 370.00 | | 361.89 | 378.11 | | <0.001 |

Abbreviations: SVB, spontaneous vaginal birth; IVB, instrumental vaginal birth; EC-L, emergency cesarean after labor started; EC-NL, emergency cesarean not in labor; ELC, elective cesarean; OB/GYN, obstetrician/gynecologist;

^a^ Spontaneous vaginal birth includes all noninstrumental vaginal births independently of spontaneous or induced onset of labor.
